# Inadequacy of nutrient intake among adolescent girls in south central Ethiopia

**DOI:** 10.1017/jns.2021.62

**Published:** 2021-10-07

**Authors:** Beza Yilma, Bilal S. Endris, Yalemwork G. Mengistu, Binyam G. Sisay, Seifu H. Gebreyesus

**Affiliations:** Department of Nutrition and Dietetics, School of Public Health, Addis Ababa University, Addis Ababa, Ethiopia

**Keywords:** 24-h dietary recall, Adolescent, Ethiopia, Micronutrient intake

## Abstract

Adolescent undernutrition is a major public health problem in Ethiopia. Inadequate dietary intake of nutrients is the major determinants of undernutrition. However, the adequacy of dietary intake among adolescents was not sufficiently explored. The present study aims to estimate the inadequacy of nutrient intake among adolescent girls in south central Ethiopia. A community-based cross-sectional study was conducted. We assess food and nutrient intake using repeated multiple-pass 24-h dietary recall. The study was conducted in Damot Gale district, Woliyta zone, Southern Ethiopia. Data were collected from 288 female adolescents. The majority of adolescent girls consumed cereals (96⋅9 %) and roots/tubers (75⋅3 %). However, only less than 1 % of them consumed flesh food. The mean energy, carbohydrate, protein and dietary fibre intake of the adolescent girls per day was 1452⋅7 ± 356⋅3 kcal, 305⋅6 ± 72⋅4 g, 35⋅7 ± 13⋅3 g and 18⋅6 ± 8⋅4 g, respectively. The median fat intake was 13⋅3 g (IQR 8⋅8, 19⋅8). The contribution of carbohydrate, protein and fat for the total energy was 80, 10 and 8 %, respectively. The prevalence of inadequate intake of protein was 60⋅9 %. The prevalence of inadequate intake of iron for early adolescents and late adolescents was 82 and 53 %, respectively. The prevalence of inadequate intake of folate was 83⋅9 % and zinc was 58 %. The prevalence of inadequate intake was greater than 90 % for vitamin B12, vitamin C and calcium. The present study found an alarmingly high prevalence of inadequate intake of some nutrients among adolescent girls of Damot Gale district.

## Introduction

Adolescents are nutritionally vulnerable due to the rapid changes in growth, development change in way of life and dietary pattern that impact both dietary intake and nutrient requirement^(1)^. It is a period where they reach nearly 15 % of their ultimate adult height, nearly half of their adult skeletal mass and half of the optimum adult weight^(2)^. At the height of their growth spurt, adolescents need a high amount of macro and micronutrients. The calcium, iron and zinc requirements double during adolescence^(2)^.

Adolescence undernutrition has been linked to delayed growth spurt^(1)^ and increases the risk of morbidity and mortality^(3,4)^. Ensuring that adolescent girls are nutritionally fit before they become mothers is vital to disrupt the vicious cycle of malnutrition. For many adolescents in developing countries, inadequate dietary intake of nutrients is one of the primary causes of malnutrition^(5)^.

The sources of energy for adolescents in middle- and low-income countries particularly for adolescents from low socioeconomic status were restricted to a repetitive consumption of limited staples^(6–8)^. The Ethiopian national food consumption survey also reported that a monotonous diet in households was common across different regions^(9)^. The consumption of calories was reported to be insufficient for the majority of schoolchildren and adolescents in developing countries^(10)^. Studies conducted in a peri-urban setting in Kenya, Bahraini and Ghana reported that only 17⋅3–64 % among school-age children and adolescents receive adequate calories^(11–13)^.

Micronutrient intake among adolescents in low and middle-income countries is generally suboptimal^(14)^. For instance, inadequate vitamin A intake among Ethiopian schoolchildren was 85 %^(15)^. In Cameroon, the prevalence of inadequacy for vitamins ranged from 20 % for vitamin A to 80 % for folate, particularly among girls^(16)^. In adolescents, an inadequate intake of vitamin B12, folate and vitamin A was reported to be 83⋅9, 81 and 45⋅3 %, respectively, while on the other hand^(17)^ in Uganda, the average intakes of calcium and zinc were 56 and 70 % of the RDA^(18)^. In Libyan schoolchildren, calcium and iron intakes were 56 and 70 % of the RDA, respectively^(19)^. Even though in some cases iron intake was adequate, majorly it is from plant sources^(20)^ with limited bioavailability. The recommended dietary allowance for protein, iron, calcium and zinc among adolescents depends on age and sex.

Studies conducted in some parts of Ethiopia showed that Stunting, thinness and anaemia among adolescents are major public health problems^(21–23)^. The Ethiopian government has developed the national nutritional programme (NNP) and set a target to prevent and control malnutrition. One of the strategic objectives of this programme is to improve the nutritional status of adolescents^(24)^. Even though the government is committed to alleviate adolescent malnutrition in all its forms, there is a lack of information to deliver targeted evidence-based intervention in regard to the intake of nutrition quality and quantity. Hence, the present study aims to estimate the inadequacy of nutrient intake among adolescent girls in south central Ethiopia.

## Methods

### Study design and study area

A community-based cross-sectional study was conducted among adolescent girls from March 2017 to April 2017 in Damot Gale district. The district is located in Woliyta Zone, Southern Nations, Nationalities and Peoples’ Region of Ethiopia.

Damot Gale district has thirty-four kebeles (twenty-eight highlands, three lowlands and three semi-urban). According to the 2007 Census, a total of 19 529 adolescent girls aged 10–19 years reside in the district. The main financial activities are agriculture and livestock rearing. Maize, enset (*Ensete ventricosum*) and root crops are the commonest staples.

### Sample size and sampling technique

The sample size was determined by using a single population proportion formula based on the following assumptions. The standard normal score set at 1⋅961 (95 % confidence interval), 5 % margin of error, 1⋅5 design effect and 10 % non-response rate.

**Table:** 

Prevalence of inadequate intake for protein (India)^(20)^	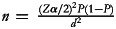
*P* = 80⋅2 %	=246
Prevalence of inadequate intake for iron (India)^(20)^	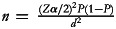
*P* = 86⋅7 %	=174

The larger sample size (246) was taken, and considering 10 % none response rate, the final sample size was 271.

A multi-stage sampling procedure was employed to identify study participants. Damot Gale district has thirty-four kebeles, of which three are urban, three lowland and twenty-eight highlands. From these kebeles, one urban, one low land and four highland kebeles were selected randomly. A simple random sampling method was employed to select study participants from selected kebeles. A list of households that have adolescent girls were obtained from the health posts through the help of health extension workers.

### Dietary assessment

An interactive, multiple-pass 24-h dietary recall questionnaire^(25)^ was used. The questionnaire was translated to the local language.

Data on commonly eaten foods, ingredients and cooking methods were collected from six households before the survey. Pictures of utensils such as bowels, spoons and cups that are commonly used to serve foods from the households of the study area were obtained. Similar utensils were purchased from the local market. The purchased utensils were calibrated by standard measuring cup and water by using a dietary scale obtained from Ethiopian Public Health Institute. The drinking cups, glasses, spoons and ladles were labelled into three categories based on their size (large, medium and small).

Moreover, a list of foods commonly consumed in Damot Gale district in the study period was prepared. The list was used as a checklist during data collection which was read to the participant after completing dietary recall to help adolescent girls recall any food they forgot to list.

Six female data collectors who had received 4 d of training collected the data. Two supervisors have supervised the data collection.

Repeated interactive 24-h dietary recall was conducted using the multiple-pass technique. In the first pass, a quick list of all foods, drinks, and snacks consumed from midnight of the previous day to midnight of the following day was obtained. In the second pass, detailed descriptions of foods and drinks consumed, including cooking procedures and brand names, were collected. Moreover, the respondents were asked if they recall any foods or drinks consumed but not included in the first pass. In the third pass, respondents were made to put the quantity of food that is nearly equal to the amount consumed on the weighing scale. For foods consumed by people, participants were made to approximate the portion they eat using utensils or salted replicas. For purchased foods, the make, name and amount were noted along with the frequency of consumption. The respondents were also asked if there was a leftover and if so, it was estimated. In the fourth pass, every respondent was asked for snacks, drinks and foods and drinks consumed outside of home of any kind.

To obtain quantity and nutrient values of purchased food market surveillance was conducted in the study area. After completing data collection, their nutrient label was used to analyse their nutrient composition.

The sample size was equally distributed to all days of the week to capture changes in intake across various days of the week. The dietary data collection was repeated in 20 % of selected adolescents by a different interviewer and on a different day from the first interview. The recall was repeated to account for the day-to-day difference in nutrient consumption of participants. The dietary data collection was not conducted on holidays or fasting days.

### Data quality management

Data collectors were trained on data collection procedures for 4 d. The primary investigator and supervisors have checked the completeness and consistency of the questionnaire. A pre-test was conducted on fourteen adolescent girls who are comparable to the actual study participants in sampled kebele. After the pre-test, we added a drinking cup that was different from the size we had.

### Data analysis

The socio-demographic characteristics of participants were entered into EpiData (Comprehensive Data Management and Basic Statistical Analysis System. Odense Denmark, EpiData Association, 2010-. http://www.epidata.dk). The analysis was performed with STATA 14 (StataCorp. 2015. Stata Statistical Software: Release 14. College Station, TX: StataCorp LP). Descriptive statistics such as frequencies, proportions, median, interquartile range (IQR), mean and standard deviation (sd) were used to describe the data. We visually inspect the histogram to assess whether continuous variables were normally distributed or not, normally distributed data were presented as mean and sd. Whereas continuous variables that are not normally distributed were presented as median and IQR (25th to 75th).

### Dietary data

#### Compilation of food composition table

To calculate nutrient values of foods, a combination of four food composition tables was used. Ethiopian food composition table^(26)^ was used to get the energy and nutrient content of foods. Some foods consumed by the adolescent girls, values of folate and vitamin B12 were missing on Ethiopian food composition table. The missing foods and nutrients were taken from USDA, West African and Tanzanian food composition tables^(27,28)^.

#### Calculating nutrient content of food

The compiled dietary data were entered into the NutriSurvey software package (NutriSurvey for windows. Copyright 2007. Dr. Juergen Erhardt SEAMEO-TROPMED RCCN, Indonesia. www.nutrisurvey.de) to create a dietary database. Then the dietary data were entered into this software to calculate the nutrient composition of the food. The data were analysed and converted to quantify nutrients and energy consumed by an individual.

#### Identifying usual nutrient intake and prevalence of inadequate nutrient intake

The within-person day-to-day variation of dietary intake was adjusted by taking into account the second-day dietary data using the IMAPP software (Intake Monitoring and Planning Program, World Health Organization, Geneva). The prevalence of inadequate vitamin B1, vitamin B2, vitamin C, folate, calcium and zinc was determined. When the prevalence of inadequacy was estimated, the IMMAP software has been adjusted for the age of participants, accounting for the variability in the recommended dietary allowance by age.

To take into account, the variation in bioavailability of iron from different food sources, absorbed iron was calculated based on the bioavailability factors of iron; 5 % of iron bioavailability was used because the diet of the adolescent girls was mainly plant-based. Values of zinc were imputed as ‘unrefined’ in the IMAPP software to account for 15 % bioavailability. Folate intake was expressed as dietary folate equivalent in the IMAPP software when calculating inadequacy.

The prevalence of inadequate intake was assessed by the percentage of adolescent girls with consumption that falls below the estimated average requirement (EAR) cut-point method except for iron. A probability approach was utilised to calculate inadequate intake of iron, since the symmetry of the need distribution did not occur.

Inadequate intake of iron was calculated by obtaining *Z*-value using the formula:
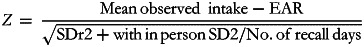


The ‘mean observed intake’ is the mean intake of iron in grams among adolescent girls.

EAR was taken from the FAO/WHO recommendation for each group of adolescent girls (early and late). SDr2 is the variance of the recommended intake (we took 10 % of the recommended intake). SD2 is the variance of intake of iron among adolescent girls. The ‘number of recall days’ was two. After obtaining the *Z*-value, we found the prevalence of inadequate intake of iron from the Z table.

#### Ethical standards disclosure

The present study was conducted according to the guidelines laid down in the Declaration of Helsinki, and all procedures involving research study participants were approved by the ethical review board of Addis Ababa University (Beza_yilma/2017). Written informed consent was obtained from all subjects greater than or 18 years old and from parents/guardians of those under 18 years.

## Results

Two hundred eighty-eight adolescents were interviewed along with their mothers or caregivers with a 100 % response rate. A second round of the 24-h dietary recall was conducted among fifty-two adolescents. One interview was dropped because of an incomplete recipe form which makes it a total of 339 interviews.

The mean and sd of adolescent girls’ age was 13⋅8 ± 2⋅2 years. Almost all of the adolescent girls (97⋅2 %) were never married. The majority of adolescent girls (84⋅4 %) were attending primary school. Most adolescent girls reside in rural areas. (Table 1)

**Table 1.** Socio-demographic characteristics of the study participants in Damot Gale district, SNNP, 2017 (*n* 288)

Characteristics	Frequency	Percent
Respondents status		
Housewife	8	2⋅8
Daughter	274	95⋅1
Relative	6	2⋅1
Residence		
Rural	240	83⋅3
Semi-urban	48	16⋅7
Respondent's age		
10–14	171	59⋅4
15–19	117	60⋅9
Education status		
In school	230	79⋅9
Out of school	58	20⋅1
Highest level of education		
Cannot read and write	4	1⋅4
Primary	243	84⋅4
Secondary	41	14⋅2
Marital status		
Never married	280	97⋅2
Currently married	8	2⋅8
Religion		
Orthodox Christian	63	21⋅9
Protestant	164	56⋅9
Muslim	8	2⋅8
Catholic	26	9
Others	27	9⋅4
Occupation		
Student	234	81⋅2
Merchant	30	10⋅4
Private business	17	5⋅9
Housewife	6	2⋅1
Others	1	0⋅4

### Foods and drinks consumed

**Fig. 1.**
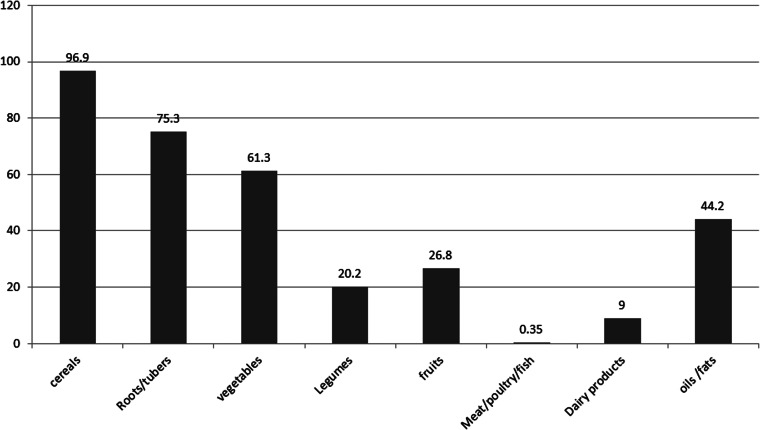
Proportion of food group consumed by adolescent girls of Damot Gale district, SNNP, 2017.

Plant-based foods such as cereal (96⋅9 %), leafy vegetables (61⋅3 %), and roots and tubers (75⋅3 %) were the most consumed food groups. However, flesh foods (0⋅35 %) and dairy products (9 %) were the least consumed food groups. Most of the adolescent girls (96⋅7 %) consumed coffee drinks (coffee beans and leaves) with their meals (Fig. 1).

### Nutrient intake of adolescent girls

The mean energy intake was 1452⋅7 ± 356⋅3 kcal/d. The contribution of carbohydrate, protein and fat for the total energy was 80, 10 and 8 %, respectively.

The mean intake of carbohydrates was 305⋅6 ± 2⋅4 g, which accounted for about 80 % of the total diet. The mean intake of protein among the adolescents was 33⋅57 ± 13⋅3 g, which was around 10 % of their diet. The median total fat intake was 13⋅4 g (IQR 8⋅8, 19⋅8), which accounted for 8 % of their diet. The median intake of vitamin A was 1952⋅8 IQR (99⋅1, 2737⋅9). Whereas the intake of dietary fibre was 18⋅6 ± 13⋅3 (Table 2).

**Table 2.** Nutrient intake of adolescents of Damot Gale district, SNNP, 2017 (*n* 339)

Nutrient	Mean	sd	Median	IQR (25th to 75th)	Contribution to total energy (%)
Energy (Kcal)	1452⋅7	356⋅3	–	–	
Protein (g)	35⋅7	13⋅3	–	–	10
Fat (g)	–	–	13⋅3	(8⋅8, 19⋅8)	8
Carbohydrate (g)	305⋅6	72⋅4			80
Dietary fibre (g)	18⋅6	8⋅4	–	–	–
Vitamin A (retinol mcg)	–	–	1952⋅8	(99⋅1, 2737⋅9)	–
Vitamin B1 (mg)	0⋅4	0⋅3	–	–	–
Vitamin B2 (mg)	0⋅7	0⋅3	–	–	–
Folate (mcg)	262	113	–	–	–
Vitamin B12	0⋅1	0⋅5	–	–	–
Vitamin C (mg)	13	11	–	–	–
Calcium (mg)	432	230	–	–	–
Zinc (mg)	11	5	–	–	–

### Prevalence of inadequate intake of nutrients among female adolescents

Nearly all adolescents are consuming inadequate vitamins C (98⋅8 %), B12 (98⋅8 %) and calcium (98⋅4 %). Similarly, 83⋅9 and 86⋅9 % of adolescents were consuming inadequate intake of B12 and folate, respectively. The prevalence of inadequate intake of protein was 83⋅9 %. The prevalence of inadequate intake of iron was 82 and 53 % for early adolescents (10–14 years) and late adolescents (15–19 years), respectively. (Table 3)

**Table 3.** Prevalence of inadequate intakes among adolescent girls (10–19 years) of Damot Gale district, 2017

Nutrient	EAR	Prevalence of inadequate intake (%)
Protein (g)	38	60⋅9
Vitamin B1 (mg)	0⋅9	93⋅7
Vitamin B2 (mg)	0⋅9	86⋅9
Folate (mcg)	330	83⋅9
Vitamin C (mg)	56	98⋅8
Calcium (mg)	1100	98⋅4
Zinc (mg)	11	57⋅9
Vitamin B12	2	98⋅8
Iron (early adolescents)		82
Iron (late adolescent)		53

EAR, estimated average requirement.

## Discussion

Identifying the specific nutrient inadequacy is a vital step to provide evidence-based intervention to alleviate the burden of malnutrition. Thus, the present study estimates the inadequacy of nutrient intake among adolescent girls in south central Ethiopia. The present study revealed a high prevalence of inadequate intakes of both macro- and micronutrients among adolescent girls in the study area. The present study also identified that flesh foods are rarely consumed by adolescent girls. The present study also showed that cereals (mainly maize) and roots/tubers (‘enset’, potato, sweet potato and yum) were highly consumed food groups among adolescent girls.

Cereals (mainly maize) and roots/tubers (‘enset’, potato, sweet potato and yum) were majorly consumed food groups and the main sources of carbohydrates and dietary. A similar finding conducted on adolescents has identified that cereals are the predominantly consumed food groups^(29)^. The 2013 Ethiopia national food consumption survey 2013 indicated that roots and tubers contributed higher proportions of foods consumed by women and children in SNNP than in other regions^(9)^.

The present study identified that the consumption of flesh foods was almost nil. This finding was comparable to the Ethiopia National Food Consumption Survey^(9)^. A study conducted in the North Shewa zone, central Ethiopia, also identified that flesh foods were seldomly consumed by adolescents^(29)^. Even though the country had a large livestock population, Ethiopia's per capita meat consumption is low. This might be due to low individual earning, personal use-focused animal rearing practices and expensiveness of meat^(30)^. Flesh foods are important sources of iron, zinc, selenium and vitamin B-12^(31)^; this very low intake of flesh foods may predispose adolescents to deficiency of these micronutrients when combined with the increased requirements during this age group.

The present study found that the total energy contribution of carbohydrate, protein and fat was 80, 10 and 8 %, respectively. The contribution of carbohydrate to total energy intake was found to be higher than acceptable limits set by the US Institute of Medicine for carbohydrates. However, it was below the limit for protein and fat^(32)^. A systematic review conducted among adolescent girls in developing countries reports similar findings that carbohydrates contribute the largest share of the total energy intake of adolescents^(33)^. Except for carbohydrate, these finding was lower than the Ethiopian national food consumption survey among other age groups^(9)^.

The prevalence of inadequate intake of iron was 82 and 53 % for adolescents and years respectively. This might be due to the low consumption of animal source foods and vitamin C reach fruits. Similarly, the intake of iron among adolescents in low and middle-income countries is low^(17)^. Adolescents from low-income countries experience monotonous or less diversified types of diets dominated by cereals, whole grains, roots and tubers; these plant-based staple foods, which are unrefined, are rich in phytates that can bind and significantly reduce the absorption of non-hem iron by forming insoluble Fe complexes. Unleavened bread made of unrefined maize flour was commonly consumed among the adolescents of the study area Cereals, such as whole-maize flour, containing 1–2 % phytic acid^(34)^. Vitamin C from fruits and vegetables is important in enhancing the absorption of non-haem iron from plant-based diets^(35)^. However, in the present study, only 0⋅35 % of adolescent girls consume meat, poultry and fish. The prevalence of inadequacy for vitamin C was found out to be 98⋅8 %. This high inadequacy could be due to low dietary intake of citrus fruits and vitamin C-rich foods. The inadequacy of Vitamin C decreases the absorption of non-haem iron. Furthermore, most of the adolescents consumed coffee drinks with their meals. Coffee is high in Phenolic compound which has marked iron-binding properties and inhibits its absorption. Even though we did not examine the effect of coffee on serum ferritin; several studies have shown that coffee consumption is associated with low serum ferritin^(36)^. A similar study also showed that a cup of coffee reduced iron absorption from a hamburger meal by 39 %^(37)^. Moreover, coffee consumption has been associated with anaemia^(38,39)^. Calcium also interferes with iron absorption which was not a concern in the present study due to low intake of milk and milk products.

The present study found 83⋅9 % prevalence of folate inadequacy among adolescent girls. Studies conducted in Africa also reported a high prevalence of folate inadequacy among adolescents girls^(16,18)^. The prevalence of folate inadequacy is higher compared to the prevalence reported by a study conducted among women of reproductive age in Ethiopia^(40)^. The difference could be due to low intake of fresh green vegetables in the present study. We also observed foods consumed in the study area were highly cooked; this might result in loss of substantial amounts of folate during cooking. Making a comparison with other studies conducted in other countries is limited due to the different recommendations and criteria for adequate intake were used. However, we can observe a low intake of calcium among low-, middle-low- and middle-income countries. Similar studies conducted in Brazilian adolescents aged 10–19 years found 89 % prevalence of inadequacy^(41)^.

Adolescents gain up to nearly half of their skeletal mass during this age group. Due to this, calcium requirement is high for bone growth and skeletal development^(42)^. However, calcium was one of the nutrients with the highest prevalence of inadequacy among adolescent girls. This could be due to the low consumption of milk products which are the main sources of calcium in the present study. Similar findings have been reported by studies conducted in both developed and developing countries where the prevalence of calcium inadequacy is high^(33,43–46)^. A Malaysian cohort study done among 13 year olds showed that calcium and vitamin D were consumed the least (<50 % of the recommended nutrient intake (RNI)), for both males and females^(47)^. Adolescents had inadequate intake of calcium 71 % in Iran^(46)^. The intake of calcium was found to be significantly far below the RDA among adolescent girls of India^(44)^ and also in Uganda, the average intake of calcium was only 56 % of the RDA^(8)^. Similar studies conducted in developed countries have reported higher calcium intakes. In the USA, Harnack *et al.* showed that calcium intake in adolescents aged from 11 to 14 years was 993 mg/d^(48)^, and the mean calcium intake evaluated on adolescents aged from 11 to 18 was estimated to be 1172 mg/d^(49)^. In Spain, a study conducted in eight cities showed that the mean of calcium intake was 859⋅9 ± 249⋅2 mg/d^(50)^.

The strengths of the present study were: by distributing the sample size to all days of the week, we were able to adjust for intrapersonal variability by using the IMAPP software. We used a salted replica of staple foods and equipped each data collector with locally used calibrated utensils. The limitation of the study was since the present study utilised a cross-sectional study design, it could not assess seasonal variability in the intake of nutrients. The other limitation was self-reported dietary intake may have resulted in recall errors and underreporting, which we tried to minimise by using a multiple-pass method that is suitable for probing and by using trained interviewers the usual intake of nutrients may not be precise as some nutrients do have huge variability-with in-person variation. A second-day repeat may help but not be sufficient to avoid errors in estimating the usual intake.

In conclusion, the result indicated a high prevalence of inadequate dietary intake of both macro- and micronutrients among adolescent girls in Damot Gale district. Therefore, improving adolescent's dietary intake is a vital strategy in preventing adolescents’ malnutrition. Further study should evaluate the dietary pattern of adolescents among adolescent girls.
